# Protein secretion and associated stress in industrially employed filamentous fungi

**DOI:** 10.1007/s00253-023-12985-4

**Published:** 2024-01-10

**Authors:** Reshma Jadhav, Robert L Mach, Astrid R Mach-Aigner

**Affiliations:** 1 Institute of Chemical, Environmental and Bioscience Engineering, TU Wien, Gumpendorfer Str. 1a, A-1060 Vienna, Austria; 2 Christian Doppler Laboratory for Optimized Expression of Carbohydrate-Active Enzymes, Institute of Chemical, Environmental and Bioscience Engineering, TU Wien, Gumpendorfer Str. 1a, A-1060 Vienna, Austria

**Keywords:** Fungi, Enzymes, Protein secretion, ER stress

## Abstract

**Abstract:**

Application of filamentous fungi for the production of commercial enzymes such as amylase, cellulase, or xylanase is on the rise due to the increasing demand to degrade several complex carbohydrates as raw material for biotechnological processes. Also, protein production by fungi for food and feed gains importance. In any case, the protein production involves both cellular synthesis and secretion outside of the cell. Unfortunately, the secretion of proteins or enzymes can be hampered due to accumulation of unfolded or misfolded proteins in the endoplasmic reticulum (ER) as a result of too high synthesis of enzymes or (heterologous) protein expression. To cope with this ER stress, the cell generates a response known as unfolded protein response (UPR). Even though this mechanism should re-establish the protein homeostasis equivalent to a cell under non-stress conditions, the enzyme expression might still suffer from repression under secretory stress (RESS). Among eukaryotes, *Saccharomyces cerevisiae* is the only fungus, which is studied quite extensively to unravel the UPR pathway. Several homologs of the proteins involved in this signal transduction cascade are also found in filamentous fungi. Since RESS seems to be absent in *S. cerevisiae* and was only reported in *Trichoderma reesei* in the presence of folding and glycosylation inhibitors such as dithiothreitol and tunicamycin, more in-depth study about this mechanism, specifically in filamentous fungi, is the need of the hour. Hence, this review article gives an overview on both, protein secretion and associated stress responses in fungi.

**Key points:**

*• Enzymes produced by filamentous fungi are crucial in industrial processes*

*• UPR mechanism is conserved among many fungi, but mediated by different proteins*

*• RESS is not fully understood or studied in industrially relevant filamentous fungi*

**Supplementary Information:**

The online version contains supplementary material available at 10.1007/s00253-023-12985-4.

## Introduction

Filamentous fungi have become a remarkable part of industrial biotechnology since the nineteenth century. The first patent in the field in 1896 was made by Dr. Jokichi Takamine for the production of digestive enzymes by *Aspergillus oryzae* (Bennett and Baker 2008). In 1942, Anne Sheafe Miller suffering from a *Streptococcus* infection was saved through penicillin synthesized by *Penicillium chrysogenum*, which was discovered in 1929 by Alexander Fleming. Basically, in the first half of the twentieth century, industrial bioprocesses used fungi for the production of organic acids and antibiotics. Later, researchers developed advanced fungal systems for the production of many other primary and secondary metabolites such as vitamins and alkaloids (Baker et al. 2008; Grigoriev et al. 2011). It is estimated that fungi are used today to make more than 700 commercial products. For instance, citric acid produced on an industrial scale by *A. niger* is used in food, soft drinks, cosmetics, and leather manufacture. Fumaric acid used in making alkyd and wetting agents is produced by *Rhizopus nigricans*. A plant growth hormone, gibberellic acid, is derived from *Fusarium moniliforme*. *Eremothecium ashbyi* is cultivated for the production of riboflavin (vitamin B2) as a vitamin supplement fat (Patel et al. 2016).

Many fungi naturally secrete a variety of enzymes in order to breakdown complex substrates into easily metabolizable mono- or oligomers or to dissolve solid substrates. Importantly, enzyme-based products and solutions are used in over 40 industry sectors such as household care, bioenergy, agriculture, animal health, and food. Food application of enzymes includes bakery, brewery, juice, wine, dairy, and oil/fats (Patel et al. 2016). Enzymes have been exploited industrially on such a vast scale due to their good performance under a wide range of physical and chemical conditions. They have several advantages over conventional methods (like chemical synthesis) such as reduced process time, cost-effectiveness, low energy input, non-toxicity, greater efficiency, higher-quality products, and eco-friendly characteristics (Gurung et al. 2013; Kamini et al. 1999; Singh et al. 2016). Application of *Aspergillus* species is of utmost importance for dairy industry as they produce acid proteinase and lipase for milk coagulation used to improve the quality of cheese (Vishwanatha et al. 2010; Neelakantan et al. 1999). Enzymes like xylanase, glucose oxidase, and protease produced by *A. niger* and *A. oryzae* play an important role in dough conditioning and strengthening, which improves the structure of bread (Saxena et al. 2001). The beverage industry employs enzymes like pectinase, amylase, cellulase, naringinase, and laccase from a number of filamentous fungi such as *A. oryzae*, *A. niger*, *T. atroviride*, *Cochiobolus miyabeanus*, and *Trametes versicolor* for processes such as depectinization, fruit liquefaction, debittering, and to synthesize aromatic aldehydes (Yamasaki et al. 1964; El-Zalaki and Hamza 1979; Singh et al. 2016; Ito and Takiguchi 1970; Fritz-Langhals and Kunath 1998). Furthermore, *Aspergillus* sp. are also employed in detergent industry to produce certain enzymes that have the capability to remove stains consisting of protein, carbohydrates, and lipids (Souza 2010; Vishwanatha et al. 2010). Leather industry utilizes enzymes like amylase, neutral protease, and lipase for fiber splitting, dehairing, soaking, and degreasing of the raw material (Souza et al. 2015; Singh et al. 2016).

According to the European Industry Association of Manufacturers and Formulators of Enzyme Products (AMFEP), the list of commercial enzymes for food, feed, and technical applications includes 243 enzymes manufactured by cultivation of microorganisms. Furthermore, over 300 food enzyme dossiers are under evaluation by the European Food Safety Authority (EFSA) and the EU FIAP (Food Improvement Agents Package) regulatory frame. Importantly, most of the commercial enzymes used in the food industry are manufactured using fungal hosts (Arnau et al. 2020). Most fungal production strains that the major enzyme companies use are recombinant, and the enzymes are mostly produced in heterologous hosts, primarily in *Aspergilli* and *Trichoderma reesei*. Though numerous attempts are made to improve the used fungal strains for enhanced production of the target enzymes and their activity, the information about the secretion mechanism and related stress is very meager among filamentous fungi (compare Supplementary Table S1). Therefore, this review article aims to shed light on the available information about protein secretion and potentially associated secretory stress that can be responsible for limiting the maximum enzyme secretion in industrially employed filamentous fungi.

## Protein secretion

Commonly, native proteins are expressed and secreted by at least 1000 times more efficient than heterologously expressed proteins in filamentous fungi (Sakekar et al. 2021). This fact led researchers to investigate the single attributes of fungal protein expression and secretion mechanisms (Sun and Su 2019). The complex and regulated process of protein production consists of three phases, synthesis, modification, and secretion. This review focuses on secretion events in filamentous fungi. To give an overview, secretion starts from translocation of the polypeptide to the ER lumen, where it undergoes folding and some post-translational modifications (e.g., glycosylation). These polypeptides are further transported to the Golgi apparatus for further modifications, followed by transport to the plasma membrane and secretion.

Although this process seems to be simple at first a glance, several steps are crucial in their details. The classical pathway for protein secretion is described in *S. cerevisiae*. It begins with targeted translocation of a polypeptide to the ER through either the signal recognition particle (SRP)-dependent or the SRP-independent mechanism. In the SRP-dependent mechanism, the secretory signal sequence (N-terminal 15–36 amino acids sequence) associated with the nascent polypeptide chain is recognized by SRPs. These SRPs direct the polypeptide to the cytosolic side of the ER where it binds to the peptide translocation complex (translocon). Here, the translocation through the ER membrane occurs co-translationally. After dissociation of the SRPs, further elongation of the peptide is carried out inside the ER lumen until the protein synthesis is completed. Then, the signal sequence is removed by a signal peptidase (Sec11, Spc1, Scp2, and Scp3) and addition of glycolipids and sugars (Antonin et al. 2000) in the ER lumen. Whereas in SRP-independent mechanism, proteins are targeted to the ER post-translationally (Conesa et al. 2001). In both mechanisms, the growing polypeptide chain interacts with cytosolic chaperones (e.g., HSP70) and co-chaperones, which subsequently engages with translocase/SEC complex (e.g., Sec62, Sec72, Sec73) in fungi and ortholog of translocon (Sec61, Sbh1 and Sss1) in *S. cerevisiae* (Romisch 1999), to enter the ER lumen (Sakekar et al. 2021). Among these, Sec61 is reported to be expressed in higher amounts during UPR, highlighting its crucial role in protein secretion (Kautto et al. 2013). Once the protein enters the ER lumen, it needs to be folded and matured to obtain its native form to become functional. This process is assisted by helper proteins named chaperones and foldases. Chaperones prevent non-productive protein–protein interactions by transiently and non-covalent bonding between non-native proteins, thus promoting correct folding (Gething and Sambrook 1992). Foldases catalyze covalent changes, such as disulfide bond formation, or proline isomerization, which are essential, but slow, and often rate-limiting steps in protein synthesis.

Now the correctly folded proteins exit the ER and enter the Golgi apparatus or a Golgi-like structure, as the classical dictyosome organization of the Golgi apparatus is not commonly present in filamentous fungi (Markham 1995). Once the vesicle arrives at the Golgi complex (Cis compartment), a tethering complex interacts with Sar1 and gets inactivated, which destabilizes the COPII coat. This reaction is called docking reaction. It generates the SNARE complex, which consists of four subunits associated with both, the vesicle and the Golgi membrane, hence facilitating the membrane fusion (Jahn and Scheller 2006; Kim et al. 2006). This membrane fusion and docking reaction are under tight regulation of the monomeric G-protein of the Rab family, Ypt1 (Novick and Zerial 1997). In the Golgi apparatus, further modifications like *N*- and *O*-glycosylation are highly conserved in fungal extracellular proteins, among which oligomannose *N*- and *O*-glycans are predominant ones (Archer and Peberdy 1997). Maras et al. (1999) have also reported the presence of glucose, sulfate, phosphate, and galactose on the linked glycans. Once the modifications in the Golgi compartment are complete, proteins are targeted either to the plasma membrane for secretion or to the vacuole.

A whole genome gene deletion strain library of *Neurospora crassa* was generated to understand the factors involved in secretion of enzymes with the focus on secretion of proteins through the fungal hyphae. The first mature leading hyphal segment of *N. crassa* has been well described with four regions; in apical (region I), sub-apical (regions II–IV), (Riquelme et al. 2002) and the protein secretion route that exists behind the apex is independent of the Spitzenkörper (Fajardo-Somera et al. 2013). This mechanism of protein secretion was presumed to be similar as the one of *S. cerevisiae* (Idiris et al. 2010; Shoji et al. 2014). The following chapter describes the knowledge about protein secretion mechanism in three industrially important filamentous fungi.

### Protein secretion mechanism in model and/or industrially employed filamentous fungi

#### Aspergillus oryzae

**Fig. 1:**
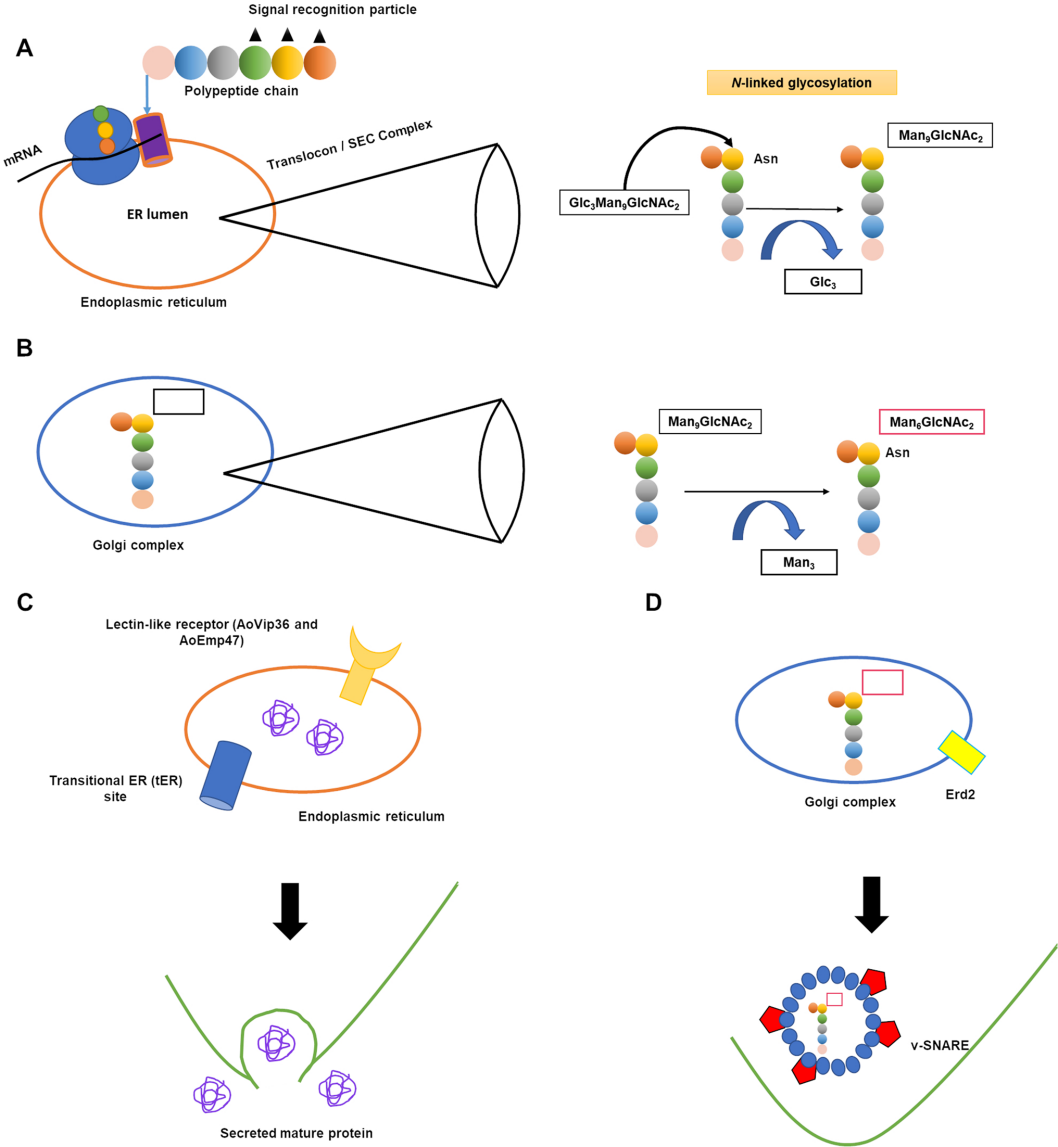
An overview of protein secretion and post-translational modifications in *A. oryzae.* **A** SRP (black triangles)-mediated polypeptide transfer into ER via translocon/SEC complex and alnexin/calreticulin cycle; Glc_3_Man_9_GlcNAc_2_ (Glc, glucose; Man, mannose; GlcNAc, N-acetylglucosamine) are attached to the asparagine (Asn) residue of glycoproteins and the glucose moiety is removed by glucosidases I and II in the ER. **B** Sequential cleavage of mannose moieties by two 1,2-α-mannosidases in the Golgi complex generate the secretory form of proteins. **C** tER- and **D** Erd2p-mediated retrieval of proteins from ER and Golgi, respectively, for subsequent secretion through the hyphal tip (green line)

In 1950s, α-amylase, historically known as Taka-amylase A from Takadiastase, encoded by three similar genes *amyA/B/C*, was isolated from *A. oryzae* (Fischer and De Montmollin 1951; Akabori et al. 1954). Due to the highest expression of *amyB* among the *amyA/B/C* genes, it was used to investigate and understand the molecular mechanism of protein secretion at the cell biology level (Tada et al. 1989; Nemoto et al. 2012). Secretory proteins harbor a signal peptide at the N-terminus targeted toward ER. And from ER, these proteins are transported to the plasma membrane via the Golgi apparatus by vesicular trafficking and eventually secreted outside the cell. While passing through ER and Golgi, the secretory proteins are modified with *N*- and/or *O*-glycan chains to provide the stability and localization (Goto 2007; Deshpande et al. 2008). In the ER lumen, during calnexin/calreticulin cycle, high-mannose oligosaccharide and a polysaccharide derivative (Glc_3_Man_9_GlcNAc_2_) are attached to the asparagine residue of glycoproteins and glucose moieties are removed by glucosidases I and II (Fig 1a). This step acts as quality control system of the glycoproteins before transporting them to the Golgi apparatus, as studied in *A. oryzae* (Watanabe et al. 2009, 2007). Thereafter, a sequential cleavage of mannose moieties by two 1,2-α-mannosidases (ManE and FmanIB) occurs at the ER and the Golgi apparatus as well, also studied in *A. oryzae* (Yoshida et al. 2000; Akao et al. 2006, 2012). The remaining Man_6_GlcNAc_2_ moiety is a secretory form of the *N*-glycan chain (Fig. 1b). Here, the single GlcNAc moiety is predicted to be important to maintain the proper protein structure and function (Kasajima et al. 2006).

Several studies over decades using state-of-the-art approaches like in vivo imaging with fluorescent proteins revealed that secretory proteins are present at the apical vesical cluster, known as Spitzenkörper, and are secreted via hyphal tips (Masai et al. 2003; Kimura et al. 2010). For septum-directed secretion a different molecular machinery is employed, which is only dependent on microtubule cytoskeleton, in contrast to hyphal tip secretion, which is dependent on actin and microtubule cytoskeleton (Hayakawa et al. 2011).

The intracellular dynamics of secretory proteins begins with correct folding in the ER lumen with the help of chaperon proteins such as BipA (binding protein). In Maruyama et al. 2006, Maruyama and co-worker reported crowded, mesh-like structures with dynamic mobility in the tip region of hyphal cells in *A. oryzae*. The study of vesicular trafficking pathway revealed a site of ER membrane, called transitional ER (tER), with punctate localization toward hyphal tip. So, the tER, in addition to lectin-like receptors AoVip36 and AoEmp47, localized to ER-Golgi are responsible for retaining secretory proteins in the ER and Golgi (Fig. 1c) (Kimura et al. 2010; Hoang et al. 2015). Further, an ortholog of Erd2p (*S. cerevisiae*) in *A. oryzae* is essential for retrieving and secretion of proteins from the Golgi apparatus (Liu et al. 2014). Now the proteins are encapsulated and transported properly, wherein vesicule-SNARE (soluble *N*-ethylmaleimide-sensitive factor attachment protein receptors) and target-SNAREs (t-SNAREs) play an important role. A complex consisting of three t-SNAREs and one v-SNARE facilitates the fusion between the membrane of secretory vesicle containing protein and the membrane of the target organelle or plasma membrane (Fig. 1d) (Rothman and Warren 1994).

Another interesting observation was that although all the proteins bearing signal peptides are destined to be secreted from the hyphal tip, certain proteins, such as the acyl-CoA binding protein AoAcb2 (Acb1 ortholog in *S. cerevisiae*), lack these signaling peptides and undergo a process called unconventional protein secretion (Malhotra 2013; Zhang and Schekman 2013).

#### Aspergillus niger

An important factor equivalent to the small GTP-binding protein Sar1p in *S. cerevisiae* was cloned and characterized in *A. niger*. It was reported to affect the protein secretion pathway, to be precise, the transport from the ER to the Golgi apparatus (Nakano et al. 1988; Nakano and Muramatsu 1989). The *A. niger* secretion-associated and Ras-related (*sarA*) gene has five introns and the encoded protein shares 70–80% identity with Sar1. Complementing *S. cerevisiae sar1* and *sec12* mutants with expression vectors containing *A. niger sarA* cDNA confirmed its functional homology. Another study using site-directed mutagenesis of *A. niger sarA* (D29G, E109K, D29G/E109K) resulted in a thermosensitive dominant-negative phenotype, differing from *S. cerevisiae*, in which similar mutations yielded a thermosensitive phenotype. This suggests that the *sarA* gene plays an essential role in *A. niger* (Veldhuisen et al. 1997).

#### Trichoderma reesei

Again, the steps involved in the secretion mechanism remain conserved in *T. reesei* and begin with the translocation of the polypeptide containing a hydrophobic signal sequence at the N-terminus into the ER lumen (Kottmeier et al. 2011). A ternary complex is formed during translation that includes the nascent protein, the mRNA, and the ribosome. This ternary complex is further directed to the ER surface via the SRP, which recognizes the signal peptide. At the surface the peptide is transported to the ER lumen through a protein channel across the ER membrane, called translocon (Corsi and Schekman 1996). The translocon in *T. reesei* consists of three subunits: Sec62, Sec72, and Sec73 (Saloheimo and Pakula 2012), that are orthologs of Sec61, Sbh1, and Sss1 present in *S. cerevisiae* (Romisch 1999). Further assistance is provided by chaperones such as Kar2, Mpd1, Mpd2, Eug1, Cpr5, and Cne1, to give the polypeptide chain its native structure (Normington et al. 1989; Frigerio and Pelham 1993; Parlati et al. 1995; Kimura et al. 2004; Pakula et al. 2003). In the ER of yeasts, during post-translational modifications, proteins are allocated with *N*-linked and/or *O*-linked glycans (Kruszewska et al. 2008; Mora-Montes et al. 2009) and such modifications were observed on secreted glycoproteins of *Trichoderma* as well. The modifications for *O*-linked glycans present in glycoproteins of *Trichoderma* include either glucose and galactose or phosphorylated or sulfonated mannobiosides and mannotriosides (Kruszewska et al. 2008). When *N*-linked glycan is present in the secreted glycoproteins, the *N*-linked glycan core possess phosphorylated mannoses as small outer chains (Kruszewska et al. 2008). These modifications are crucial for the secretory proteins as the deletion of *pmt1* (encoding a relevant protein for modifications like O-glycosylation, but not N-glycosylation) in *T. reesei* leads to the loss of a significant amount of secretory proteins (Górka-Nieć et al. 2008; Zembek et al. 2011). The next step, the packaging of proteins in coat protein complex II (COPII)-coated vesicles to facilitate the transport from the ER to the Golgi apparatus (Hernández-Chávez et al. 2014), is well described in yeast. A bioinformatics analysis of *Trichoderma* genomes has shown the presence of the genes to generate COPII-coated vesicles for this kind of anterograde transport from ER to Golgi complex (Saloheimo and Pakula 2012). But the steps following COPII-coated vesicle formation remain to be determined in filamentous fungi. The gene-encoding functional equivalents of the small GTP-binding protein Sar1p in *S. cerevisiae*, which was reported to affect the transport from the ER to the Golgi apparatus (Nakano et al. 1988; Nakano and Muramatsu 1989), were cloned and characterized in *T. reesei*. The *T. reesei* gene *sar1* has four introns, and the first intron is positioned similarly as the single *S. cerevisiae sar1* intron. The encoded protein of *T. reesei* shares 70–80% identity with Sar1 from *S. cerevisiae*. Complementing *S. cerevisiae sar1* and *sec12* mutants with expression vectors containing *T. reesei sar1* cDNA confirmed their functional homology (Veldhuisen et al. 1997).

Again, once the modifications in the Golgi apparatus are complete, the final protein with secretory *N*-glycan chain, proper protein structure, and function is trafficked toward the cell wall of mycelium or hyphae. In *T. reesei*, genes *snc1*, *sso1*, and *sso2* have been isolated and characterized. Among which products of *sso1* and *snc1* were found in subapical areas of the hyphal plasma membrane instead of apical region along with vesicle-containing Snc1 localized within the Spitzenkörper (Valkonen et al. 2007). Additionally, a complex containing Snc1 and Sso1 was also detected in the plasma membrane and compartments of the subapical region, while the complexes of Snc1 and Sso2 were found exclusively in growing apical compartments (Valkonen et al. 2007).

In 2016 another study by Nykänen and co-worker shed light on the structure of the early secretory pathway in the *T. reesei* hyphae, comparing a wild-type strain (QM6a), a cellulase-overexpressing strain (Rut-C30), and a Rut-C30 strain overexpressing a BiP1-VenusYFP fusion protein. Using various microscopic techniques, the researchers observed differences in the organization of the ER among the three strains after 24 h of growth in both cellulase-inducing and non-inducing media. The wild type showed distinct ER subdomains, including ER whorls and autophagy vacuoles, while Rut-C30 strains displayed parallel tubular/cisternal ER with fewer autophagy vacuoles and increased localization of the recombinant BiP1-VenusYFP fusion protein. The findings suggest that these structural differences are inherent traits rather than responses to protein overload in the high-secreting strain (Nykänen et al. 2016). Hence, one needs to consider the inherent properties of the particular strain when producing heterologous proteins and to not solely focus on protein overload or misfold as one of the reasons for any decrease in protein secretion.

In support of the above statement, a study by Nykänen and co-worker in 1997 was the first to explore how a fungus simultaneously produces and secretes both a native protein (the cellobiohydrolase I (CBHI)) and a foreign/heterologous protein (the barley endopeptidase B (EPB)). They found that the locations where these proteins are made (translational sites) match where their genetic instructions are read (transcriptional locations). However, the native CBHI is produced and secreted throughout the mycelium, while the foreign EPB is only secreted via specific parts, i.e., at apical and subapical cells. This suggests that CBHI might have a signal-promoting effective secretion. Comparing the transcript levels of the immunoreactive EPB and the native CBHI revealed that the translation efficiency of the recombinant mRNA was quite high. Therefore, the low yields of the secreted recombinant EPB cannot be attributed to translation issues. The study also discovered that the efficiency of producing the foreign EPB is reasonably high, so low yields are likely due to factors like degradation or incomplete processing rather than translation problems. Further research would be required for understanding how EPB is processed in the fungus (Nykänen et al. 1997). Hence, there is a still a need to study more in detail how the heterologous proteins are processed in filamentous fungi.

## ER stress

Unfolding or misfolding of proteins accounts for a significant threat to all living cells. Proteins can be unfolded or misfolded in several subcellular compartments such as cytoplasm, peroxisomes, and mitochondria. But the risk of protein misfolding is particularly acute in the ER. Here, newly synthesized secretory and transmembrane proteins are transformed into proper tertiary structures. Even though eukaryotic cells have efficient quality control systems, which evolved over time to prevent incompletely folded molecules, the accumulation of misfolded proteins in the ER effects detrimentally the function and/or localization of approximately one-third of all cellular proteins. There are three different mechanistic levels to deal with the arise of unfolded proteins, namely, transcriptional induction, translational attenuation, and degradation (Mori 2000). The coordination between these mechanisms improves the efficiency of folding, processing, and exporting of secretory proteins. Additionally, it also removes the fraction of polypeptides that failed to fold and are responsible for reduced flow of proteins into the ER compartment. The intracellular signaling pathway, common from yeast to human, starts in the ER and ends at nucleus, is called UPR. The UPR target genes mostly encode for chaperons (Bip/Grp78 and Grp94) and enzymes involved in protein folding in the ER (protein disulfide isomerase encoded by *pdi1* and peptidyl-prolyl cis-trans isomerase). The present literature depicts that the target genes also include numerous proteins involved in several steps or stages of the secretion pathway (Travers et al. 2000). The UPR itself includes three signaling pathways mediated either by IRE1 (inositol-requiring enzyme 1), PERK (protein kinase R-like ER kinase), or ATF6 (Activating transcription factor 6) that plays a vital role in re-establishing protein homeostasis of the cell. Among these, the Ire1 pathway is studied most extensively as it is the only one present in *S. cerevisiae*. It involves spliceosome-independent splicing of *hac1* and *xbp1* mRNA in yeast or mammalian cell, respectively. Recent evidence suggests that the unspliced mRNA in mammals is actually translated, and a hydrophobic segment in the nascent protein helps to target the mRNA to the ER membrane, thereby facilitating its splicing (Yanagitani et al. 2009). On the contrary, the long intron of 252 nt in the *S. cerevisiae Hac1* blocks the translation of the unspliced mRNA by forming a stem-loop structure. This stem-loop interacts with the 5’UTR of the unspliced *Hac1* mRNA, thereby stalling ribosomes (Rüegsegger et al. 2001). The removal of this intron by Ire1-mediated splicing releases the translation block, allowing the spliced mRNA to be translated.

Several pieces of evidence have been reported on the use of transgenic UPR strains in biotechnology industry to improve enzyme production. By expressing the activated form of the transcription factor HacA, induction significantly increased the production of *T. versicolor* laccase by up to sevenfold and bovine preprochymosin by up to 2.8-fold in this biotechnologically important fungus (Valkonen et al. 2003b). As additional examples, overexpressing the transcription factor Hac1 in *A. awamori* resulted in a 7- and 2.8-fold increased production of laccase and bovine prechymotrypsin is reported, respectively (Valkonen et al. 2003b). Further, disrupting the autophagy-related gene *aoatg15* in *A. oryzae* resulted in a threefold increase in bovine chymosin secretion (Yoon et al. 2013). The following section describes the understanding of UPR and the impact of UPR on production processes in industrially employed filamentous fungi.

### Examples of UPR and RESS in model and /or industrially employed filamentous fungi

#### Aspergillus fumigatus

The *A. fumigatus* UPR initiates with the proximal ER stress sensor IreA, comprising a similar domain organization as the yeast Ire1 (Feng et al. 2011). However, the target mRNA, *hacA*^*u*^, contains a short, 20 nucleotides (nt)-long intron (Richie et al. 2009) in contrast to the 252 nt-long intron in the corresponding *hac1* mRNA in *S. cerevisiae*. The *hacA*^*u*^ intron is similar in length to what has been already described in mammals, *Candida albicans*, *Caenorhabditis elegans*, and some filamentous fungi. Due to its small size, i.e., only 20 nt in *HacA*^*u*^, it is considered not to be involved in blocking 5′UTR of the unspliced *hacA*^*u*^ mRNA (Calfon et al. 2002; Wimalasena et al. 2008; Saloheimo et al. 2003; Mulder et al. 2004). Another prominent difference between the yeast and *A. fumigatus* UPR is the size of the reading frame shift caused by removal of the unconventional intron in the *hac1/hacA* mRNAs. In *A. fumigatus*, the *hacA*^*u*^ mRNA encodes a protein of 433 amino acids. The removal of the unconventional intron results in a replacement of a C-terminus domain of 220 amino acids of the HacA^u^ protein with a 129 amino acids C-terminus domain. It was observed that high levels of exogenous ER stress (e.g., DTT) triggered the signaling through IreA-HacA pathway. This results in a signal transduction cascade influencing solely the secretory pathway elements, such as protein folding, ER translocation, vesicular trafficking, ER glycosylation, and ER degradation (Feng et al. 2011). However, in the absence of any exogenously induced ER stress, the transcriptional response of the UPR was more pronounced and more diverse in function, suggesting that the UPR is constantly modifying the output of the pathway in proportion to the stress level, even during normal growth. It is speculated that the challenge of delivering cell wall and membrane components to rapidly growing hyphal tips of fungi creates fluctuations in ER stress requiring dynamic changes in UPR activity to maintain ER homeostasis. Krishnan and co-worker made a surprising observation while studying UPR in *A. fumigatus*: a large fraction of differentially expressed genes in the absence of external ER stress were dependent on IreA, but not on HacA. This further suggests that IreA controls dual signaling circuits that can be both HacA dependent and HacA independent, particularly in this organism (Krishnan and Askew 2014).

#### Aspergillus niger

Examining gene regulation under secretion stress through genome-wide expression analysis has provided a comprehensive understanding of the same (Sims et al. 2005; Arvas et al. 2006; Guillemette et al. 2007; Carvalho et al. 2012; Kwon et al. 2012). For example, a core set of 40 genes crucial for high-level glucoamylase expression in *A. niger* was identified (Kwon et al. 2012). During ER stress induced by DTT and overexpression of human tissue plasminogen activator (t-PA) in *A. niger*, 25 genes exhibited similar upregulation (Guillemette et al. 2007).

Based on the importance of HacA in protein secretion, Valkonen and co-worker introduced a new approach to improve foreign-protein production in contrast to commonly used method of overexpression of specific chaperones and foldases. By expressing the activated form of the transcription factor HacA, they could achieve constitutive induction of the UPR pathway in *A. niger* var. *awamori*. To understand the regulatory scope of UPR, authors examined the mRNA levels of new *A. niger* var. *awamori* genes involved in various secretory functions and their findings revealed both similarities and differences compared to studies in *S. cerevisiae*. (Valkonen et al. 2003b). While there is an increase in the production of a native protein (invertase) in yeast, no such beneficial effect was observed in *A. niger* var. *awamori* on the production of native proteins. This discrepancy may be attributed to the lower secretion capacity of yeast, where subtle adjustments in the secretory machinery significantly impact the production of native proteins. Additionally, differences in the regulation of the secretory machinery between the two organisms could also contribute to this variation (Valkonen et al. 2003b). Many approaches have been employed to enhance protein production and secretion in *A. niger* based on this literature. Constitutive expression of active HacA seems adequate to reprogram the ER and downstream components of the secretory pathway. And this observation aligns with findings in *S. cerevisiae* (Valkonen et al. 2003a; Breinig et al. 2006) and *Pichia pastoris* (Vogl et al. 2014), as well as the filamentous fungus *A. niger* (Valkonen et al. 2003b), where constitutive activation of the UPR positively impacted heterologous protein production. Overexpressing UPR-related genes like *sstC*, *hacA*, *gptA*, *pdiA*, *ostA*, *cnxA*, or *eroA*, and deleting genes encoding ER-associated protein degradation (ERAD) components such as *hrdC*, *derA*, or *doaA*, also increased the production and secretion of heterologous proteins in *A. niger* (Sagt et al. 2008). Alongside UPR and ER stress-related genes, altering ERAD by deleting the factor DoaA and elevating oligosaccharyltransferase SttC (Yan and Lennarz 2002) in *A. niger* has been reported to increase the β-glucuronidase yield (Jacobs et al. 2009), given their involvement in glycosylation of secretory proteins. The investigation of the *A. niger* gene *derA* reveals that its deletion can not only enhance protein production but also impact cell growth (Carvalho et al. 2011; Richie et al. 2011). This highlights that the performance of protein secretion, regulated by UPR and ERAD, can be dependent on the host.

### Neurospora crassa

In *N. crassa*, ER stress response is studied for induction and secretion of lignocellulose-degrading enzymes (Fan et al. 2015). Under ER stress conditions, 766 genes were found upregulated, of which 223 genes were regulated by the key component of UPR, i.e., IRE-1, and 186 genes by HAC-1. A total of 249 genes appeared to be pivotal for resistance against ER stress. The authors also analyzed the transcription factor (TF) network that putatively coordinates the signal flow and gene expression during the ER stress response and cellulase synthesis. Under such conditions, 33 TFs were upregulated including the UPR regulator HAC-1 and CPC-1 (a regulator associated with amino acid biosynthesis). HAC-1 was found to act as an important factor for secretion of lignocellulose-degrading enzymes, while it does not mediate the RESS feedback loop in *N. crassa*. When there is stress in the ER, the mRNA of *hac-1* undergoes a splicing reaction (removal of 23 nt intron), thereby changing its open reading frame. Disrupting the *N. crassa hac-1* gene revealed that it is essential not only for activating the UPR but also for proper growth of the fungus when exposed to chemicals causing ER stress. When *N. crassa* was subjected to grow on cellulose, which requires the secretion of many enzymes and consequently, the UPR and ER function become very important, then growth is significantly affected without *hac-1*. However, growth on hemicellulose, another substrate demanding enzyme secretion, is not affected in the mutant, suggesting that overall secretion is not altered without *hac-1* (Montenegro-Montero et al. 2015). Further, CPC-1 may play a critical role in *N. crassa* ER stress response and cellulase secretion, although its regulation does not seem to be controlled by the IRE-1/HAC-1-mediated UPR cascade (Fan et al. 2015).

A homolog of the TF Ace1 (a cellulase repressor) in *T. reesei* and StzA (a stress responsive factor) in *A. nidulans* was identified as NCU09333 in *N. crassa* (Aro et al. 2003; Chilton et al. 2008). This TF is upregulated during ER stress; however, its role seems to be rather a stress responsive factor as it had limited effect on cellulase secretion. The expression level of the carbon catabolite repression regulator CRE-1 (NCU08807) in *N. crassa* was also found to be upregulated during ER stress. Based on experimental evidence, authors suggested that the carbon catabolite repression pathway might cross-talk with the ER stress response (Fan et al. 2015).

RESS in *N. crassa* was shown to be independent of the IRE-1/HAC-1-mediated UPR transduction cascade. RESS-mediated repression of cellulase genes was likely to be a direct result of the down-regulation of the two regulators CLR-2 and XLR-1. To support this assumption, the expression levels of some carbohydrate transporter genes belonging to the CLR-2 and XLR-1 regulons in *N. crassa*, i.e., CDT-1 (NCU00801) and LAT-1 (NCU02188), were evaluated. Both genes had a decrease in expression level; however, how it is triggered in the presence of ER stress remains to be investigated (Fan et al. 2015). Also, understanding the enzyme secretion pathway in *N. crassa*, wherein HAC-1 does not play the crucial role, will widen the knowledge about fungal mechanisms for breaking down plant cell walls, which in turn could have impact on industrial biotechnology (Montenegro-Montero et al. 2015).

#### Trichoderma reesei

**Fig. 2:**
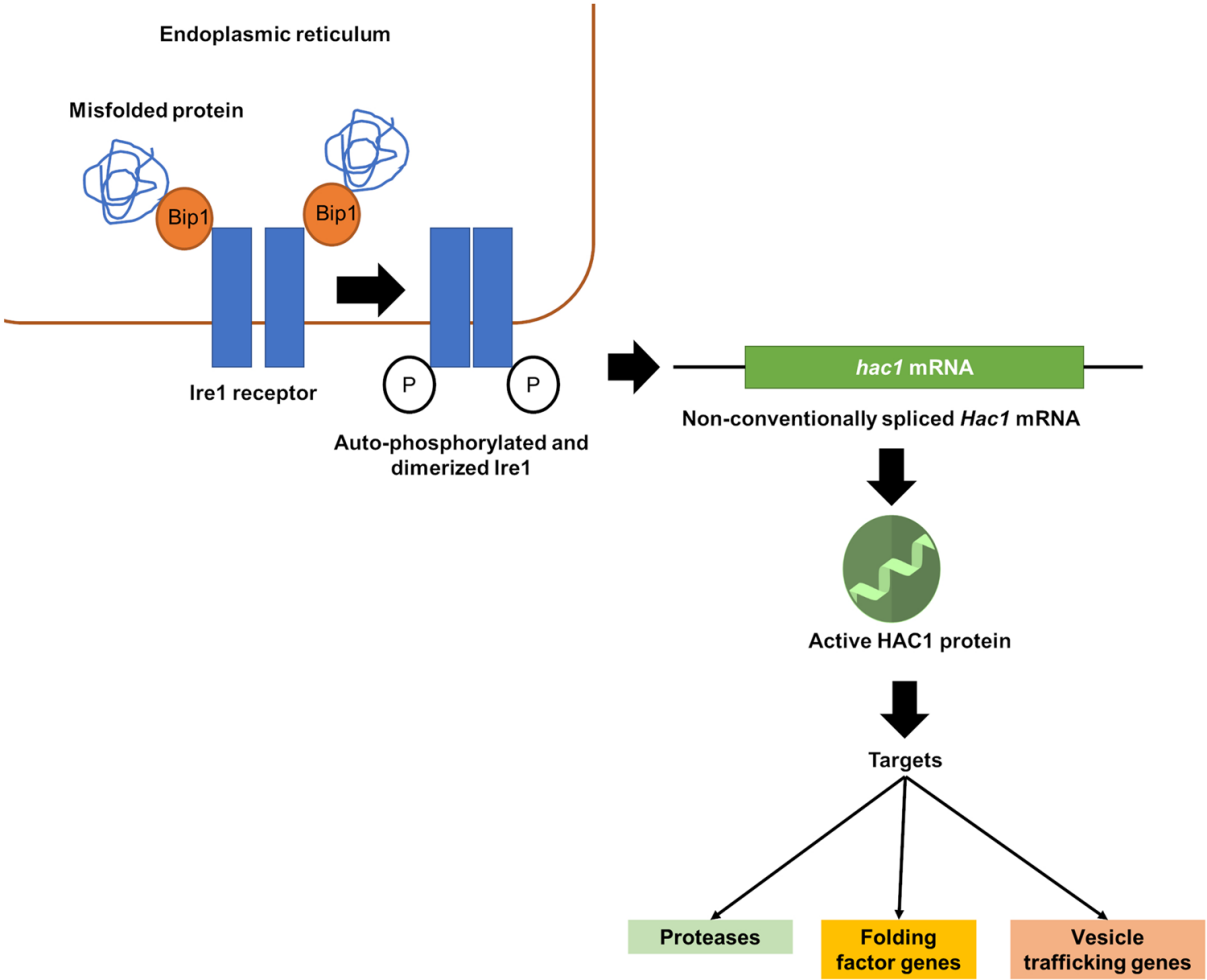
An overview of the Hac1-dependent, Ire1-mediated response to the accumulation of misfolded proteins in the ER of filamentous fungi

In case of *T. reesei*, the mechanism to cope up with ER stress remains conserved. Here also unfolded proteins bind to the ER chaperone Bip1. This hinders the binding of Bip1 to the transmembrane protein kinase Ire1. Ire1 activates itself by homodimerization and autophosphorylation and splices the mRNA of the transcription factor Hac1 (Fig 2). In *T. reesei* a short intron of 20 bp is spliced from the *Hac1* mRNA and additionally, a long part at the 5′-flanking region (Saloheimo et al. 2003). The truncation of the 5′-flanking region (250 nt) can be mediated either by ribonuclease cleavage or by transcription from a different start site. It was suggested that preferably the latter is the case in *T. reesei* because the region is extremely CT rich, which is a typical feature for a transcription start site (Gurr 1987). *T. reesei Hac1* mRNA has two upstream ORFs encoding 18 and 2 amino acids. The longer one is absent from the mRNA during the induction of UPR. The presence of 2 amino acids in the 250 nt-truncated *Hac1* indicates its non-significant effect on *Hac1* mRNA translation. Hac1 upregulates expression of chaperones, proteases, and vesicle trafficking genes (Fig. 2) that work together to remove the unfolded proteins from the ER and re-establish the balance in the protein secretion pathway (Travers et al. 2000). In a study in *T. reesei*, the results from the combined analysis of the expressed sequence tag (EST) collection of *T. reesei* subjected to different stress conditions confirmed the induction of Bip1 and Pdi1, characterized as UPR-related genes (Saloheimo et al. 2003; Pakula et al. 2003). Furthermore, ESTs corresponding to at least 457 genes were found to be putatively induced during secretion stress in *T. reesei*. A specific EST homolog present in *T. reesei* corresponds to *cpc* gene in filamentous fungi or to Gcn4 of *S. cerevisiae*, which regulates amino acid biosynthesis (Arvas et al. 2006). However, the key difference is their activation under amino acid deprivation conditions. Gcn4 is regulated at the translational level, whereas Cpc proteins are found to be controlled at both the translational and transcriptional level (Wanke et al. 1997; Paluh et al. 1988; Albrecht et al. 1998). The corresponding Cpc1 targets in *T. reesei*, that are induced or affected under UPR, are the *glt1*, *arg1*, and *aro1* genes (Arvas et al. 2006). In addition, two more crucial genes related to protein secretion, Ypt1/YptA and Nsf1/NsfA, were described in *T. reesei* and *A. niger* var. *awamori.* These genes encode for the Rab protein and the general fusion factor, respectively. Both show high conservation with their counterparts in *S. cerevisiae* and mammals. The *T. reesei ypt1* gene resembles the effect observed in a yeast Ypt1p (Ypt1 protein) deletion study. Transcriptional regulation of *T. reesei* genes involved in protein trafficking (*ypt1*, *nsf1*, and *sar1*) was examined using the protein-folding inhibitor DTT and the protein-trafficking inhibitor brefeldin A. DTT induced *nsf1* and *pdi1*, *sar1* mRNA increased under strong UPR induction, and *ypt1* mRNA did not show a clear increase. Brefeldin A strongly induced *pdi1* and other intracellular trafficking genes, suggesting a potential transcriptional activation of the entire secretory pathway of *T. reesei* in response to protein accumulation stress. This is in contrast to *S. cerevisiae*, in which only a small subset of the secretory pathway-encoding genes responds to the UPR (Saloheimo et al. 2004). Another comparative study of the homologs of the *S. cerevisiae* proteins Ire1 and Ptc2 in *T. reesei* resulted in the confirmation of the same functional activity in both organisms. The *T. reesei* Ire1 protein displays inherent kinase activity, which was demonstrated in vitro by an autophosphorylation assay. When Ire1 was overexpressed in a *T. reesei* strain producing a foreign protein (laccase 1 from *Phlebia radiata*), an up-regulation of the UPR pathway was observed. by detecting elevated expression levels of UPR target genes such as *bip1* and *pdi1* (Valkonen et al. 2003a).

Also, elevating the expression of genes encoding protein disulfide isomerase (Pdi) or Kar2/Bip was demonstrated to increase the production of certain foreign proteins in both *S. cerevisiae* and filamentous fungi (Punt et al. 1998). This study illustrated that inducing the UPR by overexpressing *S. cerevisiae hac1* and *T. reesei hac1* in *S. cerevisiae* enhanced the production of both foreign and native proteins (Valkonen et al. 2003a). In *T. reesei*, overexpressing *bip1* and *hac1* led to a 1.5- and 1.8-fold increase in the secretion of heterologous glucose oxidase (Wu et al. 2017). Furthermore, *hacA* overexpression in *A. niger* var. *awamori* stimulated the expression of two foreign proteins, *T. versicolor* laccase1 and calf preprochymosin (Valkonen et al. 2003b; Valkonen et al. 2004). In addition, Pakula et al. observed a transcriptional down-regulation of secreted protein-encoding genes under secretion stress in *T. reesei*. This response was termed as RESS, which was also described in *A. niger* and *A. nidulans* (Al-Sheikh et al. 2004; Sims et al. 2005). However, how RESS is triggered during UPR and whether it acts instead of or additionally to regulated Ire1-dependent pathway is unknown. A previous study indicated that elevated Cre1 expression levels cause perturbations in intracellular glucose homeostasis under ER stress conditions. And this observation raises the possibility that metabolic repression caused by internal fluctuations of intracellular nutritional cues (e.g., simple sugars or free amino acids) might have an impact on RESS, although more data need to be acquired to support this hypothesis (Fan et al. 2015).

## Outlook

A recent study using *S. cerevisiae* strains with W303 background (an allelic variant of *mip1*, which increases petite frequency and lacks a functional copy of the RNA-binding protein and translational repressor Ssd1) revealed that the nucleosome spacing–enzyme Isw1 (ATP-dependent chromatin remodeler), which promotes the accessibility of chromatin and exports nuclear-retained mRNPs (Babour et al. 2016), is critical to fine tune the UPR homeostasis feedback loop. It binds to the 3′UTR of the *hac1* transcript and limits its nuclear export resulting into cytoplasmic splicing (Matabishi-Bibi et al. 2022). This raises the questions firstly, about its presence in industrially important filamentous fungi, and secondly, whether it has any effect on RESS and eventually on the secretion of hydrolytic enzyme either at transcriptional or translational level.

To conclude, protein secretion is a crucial and complex process in filamentous fungi and the ER plays a central role in protein synthesis, folding, and secretion. However, the high demand for protein production can lead to ER stress, triggering the UPR. The UPR is a multifaceted adaptive mechanism that aims to restore ER homeostasis and ensure correct protein folding and secretion. It involves the upregulation of chaperones, foldases, and protein degradation. But the systematic information on ER stress at a cellular level is explored in only three model systems, mammalian cells (human B cells) (Dombroski et al. 2010), *Drosophila* lines (Chow et al. 2013), and knock-out mutants of *S. cerevisiae* (Jonikas et al. 2009). The information on ER stress along with the protein secretion pathway in industrially employed filamentous fungi (particularly enzyme producers) points to a lack of knowledge on a) association between protein secretion and ER stress, b) how antioxidants could reduce ER stress in relation with oxidative stress, and c) RESS, which might be a crucial problem for heterologous or overexpression of enzymes produced at industrial scale. The latter point is important because there are some fungi, which seem to either do not have or have Hac1-independent RESS mechanisms, like *A. fumigatus* and *N. crassa,* while others, like *T. reesei*, do. Hence, it would be beneficial to investigate more industrially employed fungi for this mechanism to reveal the precise connection to UPR that might allow to modify or inhibit RESS.

## Supplementary Information

ESM 1 (PDF 81 kb)
