# Supplementary material for: Protein secretion and associated stress in industrially employed filamentous fungi
Source: Appl Microbiol Biotechnol. 2024 Jan 10;108(1):92. doi: 10.1007/s00253-023-12985-4 (PMC10781871; doi:10.1007/s00253-023-12985-4)
Supplement: Supplementary file 1 — (PDF 81 kb) [file 253_2023_12985_MOESM1_ESM.pdf]

Applied Microbiology and Biotechnology

Protein secretion and associated stress in industrially employed filamentous fungi

Reshma Jadhav[1,2], Robert L Mach[1], Astrid R Mach-Aigner[1,2*]

[1]Institute of Chemical, Environmental and Bioscience Engineering, TU Wien, Gumpendorfer Str. 1a, A-1060 Vienna, Austria
[2]Christian Doppler Laboratory for Optimized Expression of Carbohydrate-Active Enzymes, Institute of Chemical, Environmental and Bioscience Engineering, TU Wien, Gumpendorfer Str. 1a, A-1060 Vienna, Austria

*Corresponding author: Astrid Mach-Aigner, PhD
E-mail address: astrid.mach-aigner@tuwien.ac.at

Supplementary Table S1. Overview on the availability of information on protein secretion and ER stress mechanisms in industrially employed filamentous fungi for enzymes production. IA, information is available, information is not available.

Enzyme	Microorganism	Protein secretion	ER stress	Application
Acid proteinase	*Aspergillus* sp.	IA	INA	Milk coagulation
Aminopeptidase	*A. niger*	IA	INA	Synthesis of biopeptides and amino acids
	A. oryzae	INA	INA	
AMP deaminase	*A. melleus*	INA	INA	
Amylase	*A. niger*	INA	IA	Fibre splitting, carbohydrate stain removal, used in brewing and fermentation industries, and the laundry industry
	A. oryzae	IA	IA	
	Lichtheimia ramosa	INA	INA	
	Thermoascus aurantiacus	INA	INA	
	Rhizopus oryzae	INA	INA	
Arabinofuranosidase	*A. niger*	INA	INA	Clarification of fruit juices, digestion enhancement of animal feedstuffs and as a natural improver for bread
Catalase	*A. niger*	INA	INA	Improving the quality of cheese
Cellobiase	*A. niger*	IA	INA	Hydrolysis of cellobiose
	T. reesei	INA	INA	
Cellulases	*T. reesei*	IA	IA	Fruit liquefaction, hydrolysis of cellulose
	T. atroviride	IA	INA	
	Penicillium funiculosum	INA	INA	
	A. niger	INA	INA	
	A. fumigatus	INA	INA	
	Sporotrichum thermophile	INA	INA	
Dextranase	*Chaetomium erraticum*	INA	INA	Hydrolysis of dextran
Feedase	*Penicillium funiculosum*	INA	INA	Hydrolysis of the indigestible fraction of feed
Galactosidase	*A. niger*	INA	INA	Beet sugar refining, antitumor activity
Glucanase	*A. niger*	INA	INA	Hydrolysis of the barley gums in the brewing industry
	T. reesei	INA	INA	
	Talaromyces emersonii	INA	INA	
	Humicola insolens	INA	INA	

Glucose oxidase	*A. niger*	INA	INA	Dough strengthening, oxygen removal from beer
	A. tubingensis	INA	INA	
	P. chrysogenum	INA	INA	
	Penicillium purpurgenum	INA	INA	
	P. INAtatum	INA	INA	
Glucosidase	*Myceliopthora heterothalica*	INA	INA	Metabolism of glycolipids and dietary glucosides, and signaling functions
	Gongronella butleri	INA	INA	
	F. oxysporum	INA	INA	
	Penicillium sp. *LMI01*	INA	INA	
Hemicellulase	*A. niger*	INA	INA	Modification of the structure of xylan and glucomannan in pulp fibers in order to enhance the chemical delignification
	Trichoderma sp.	IA	IA	
	Humicola sp.	INA	INA	
Invertase	*A. flavus*	INA	INA	Soft-centered candies, artificial honeys, confectioneries, liqueurs
	A. caespitosus	INA	INA	
	Cladosporium herbarum	INA	INA	
Chitinase	*T. viride*	INA	INA	Breakdown of chitin
	P. chrysogenum	INA	INA	
	T. koningiopsis	INA	INA	
Laccase	*Trametes versicolor*	INA	IA	Biodegradation, oligomerization of proteins and sugar derivatives, polymerization to form functional polymers
	Trametes villosa	INA	INA	
	Trametes hirsuta	INA	INA	
	Trametes pubescens	INA	INA	
	P. chrysogenum	INA	INA	
	Ganoderma lucidum	INA	INA	
	A. fumigatus	INA	INA	
L-asparaginase	*A. niger*<br>*A. terreus*<br>*P. digitatum*<br>*F. equiseti*<br>*Emericella nidulans*	INA<br>INA<br>INA<br>INA<br>INA	INA<br>INA<br>INA<br>INA<br>INA	Hydrolyzes L-asparagine to L-aspartic acid and ammonia, applied in pharmaceutical and food industries
Ligininase	*A. niger*<br>*P. ostreatus*<br>*Xylaria* sp.	INA<br>INA<br>INA	INA<br>INA<br>INA	Degrade a wide variety of structurally diverse organic compounds, including a

				number of environmentally persistent orgaINApollutants
Lipase	*Trichoderma* sp.<br>*Penicillium* sp.<br>*A. niger*	INA<br>INA<br>INA	INA<br>INA	Cheese ripening, flavored cheese, Fat stain removal, degreasisng
Lysophospholipase	*A. niger*	INA	INA	Processing of starch for glucose syrups production, and for degumming of fats and oils
Naringinase	*A. niger*<br>*A. oryzae*<br>*A. usamii*<br>*Rhizopus nigricans*	INA<br>INA<br>INA<br>INA	INA<br>INA<br>INA<br>INA	Debittering in beverage industry
Pectinase	*A. niger*<br>*P. funiculosum*<br>*P. oxalicum*	INA<br>INA<br>INA	INA<br>INA<br>INA	Depectinization
Peroxidase	*Mushroom*<br>*Coprinus*			
Protease	*A. melleus*<br>*A. oryzae*<br>*A. niger*<br>*A. flavus*<br>*A. flavipes*<br>*A. brasiliensis*<br>*Penicillium citrinum*<br>*Rhizomucor miehei*	INA<br>INA<br>INA<br>INA<br>INA<br>INA<br>INA<br>INA<br>INA	INA<br>INA<br>INA<br>INA<br>INA<br>INA<br>INA<br>INA<br>INA	Protein stain removal, restrict haze formation, to improve the aroma of bread, dehairing, soaking
Ribonuclease	*Penicillium citrinum*	INA	INA	Catalyzes degradation of RNA
Tannase	*A. oryzae*	INA	INA	Preparation of instantaneous tea, action on tea polypheINAls, production of gallic acid, beer chillproofing and wine making
Xylanase	*T. reesei*<br>*T. longibrachiatum*<br>*Humicola insolens*<br>*A. niger*<br>*Rhizopus oryzae*<br>*A. fumigatus*	INA<br>INA<br>INA<br>INA<br>INA	INA<br>INA<br>INA<br>INA<br>INA	Dough conditioning
